# Molecular Markers of MDR of Chemotherapy for HSCC: Proteomic Screening With High-Throughput Liquid Chromatography-Tandem Mass Spectrometry

**DOI:** 10.3389/fonc.2021.687320

**Published:** 2021-06-28

**Authors:** Bin Shen, Xuelin Dong, Bo Yuan, Zhijun Zhang

**Affiliations:** Department of Otolaryngology, Shuguang Hospital Affiliated to Shanghai University of Traditional Chinese Medicine, Shanghai, China

**Keywords:** HSCC, FADD, RIPK1, multi drug resistant, molecular markers

## Abstract

**Background:**

Hypopharyngeal squamous cell cancer (HSCC) is a head and neck tumor with a poor prognosis. Chemotherapy lacks effectiveness because of multidrug resistance (MDR), which has increased toxic side effects. Thus, there is an urgent need to identify the molecular markers of MDR of chemotherapy for HSCC.

**Methods:**

Fifty clinical samples of HSCC were derived from patients including 12 sensitive or resistant to chemotherapy drugs. Proteomic screening was performed using liquid chromatography-tandem mass spectrometry (LC-MS), which was based on data-independent acquisition. Molecular markers of MDR of chemotherapy in patients with HSCC were identified with clinical data and validated with ELISA.

**Results:**

A total of 673 differentially expressed proteins were identified in HSCC samples, where 172 were upregulated and 501 were downregulated. A total of 183 differentially expressed proteins including 102 upregulated and 81 downregulated proteins, were identified by comparing cancer sensitive to chemotherapy with cancer resistant to chemotherapy. Clinical HSCC samples had significantly higher expression of FADD and significantly lower expression of RIPK1. Expressions of FADD and RIPK1 proteins were significantly lower in the chemotherapy-sensitive group. These expression differences were not correlated with clinical data. RIPK1 and FADD are involved in necroptosis and the signaling pathway of PRRs. Using ELISA, the low expression of RIPK1 and FADD was found in the patients sensitive to chemotherapy.

**Conclusion:**

LC-MS proteomics is an effective method to identify the molecular markers of HSCC. FADD and RIPK1 can act as molecular markers of MDR of chemotherapy in patients with HSCC and may function through necroptosis and the PRR signaling pathway.

## Introduction

Hypopharyngeal squamous cell cancer (HSCC), a head and neck squamous cell cancer (HNSCCs), accounts for approximately 1.4%–5.0% of cases with head and neck malignant tumor (1). The disease progresses with lymph node metastasis in 70%–87% of patients at initial presentation because of high malignancy and insidious onset (1, 2). Furthermore, lesions are also found in the pharynx and larynx. These characteristics pose a considerable treatment challenge and make HSCC a major life-threatening illness. The 5-year overall survival of HSCC is only 30%–35% (3–5).

Most patients with HSCC currently undergo comprehensive therapy with combined chemotherapy, surgery, and radiation therapy as common clinical procedures. We previously conducted a single-center, randomized, double-blinded clinical study in patients with HSCC. Both induction chemotherapy and control groups underwent surgery and postoperative radiotherapy; the former was treated with Erbitux (cetuximab), lobaplatin, and 5-Fu. The induction chemotherapy group in comparison with the control group had significant increases in overall and partial response rates as well as elevations in larynx preservation, patient quality of life, and 5-year survival rate (P < 0.05). However, 40.62% of patients remained insensitive to targeted or chemotherapy drugs or appeared initially sensitive but were subsequently resistant to drugs; these patients did not achieve remission and some progressed to disease. In this context, although considerable therapeutic effects have been observed, platinum-based, and anti-EGFR targeted drugs still have significant shortcomings, such as multiple drug resistance that severely limits their efficacy.

Several studies have assessed possible biomarkers for HSCC. Wendt (6) proved that human papillomavirus (HPV)-induced hypopharyngeal cancer is rare and that p16 is not an appropriate biomarker of HPV in this tumor. A positive correlation between low expression of PCDH20 (as an independent prognostic factor) with T staging and lymph node metastasis has been established, although the sample size was small (7). Zhang (8) corroborated the upregulated BTF3 expression in HSCC, which is positively correlated with HSCC lymph node metastasis. Upregulated S100A4 expression in HSCC was substantiated by Xu (9) and was positively correlated with neck lymph node metastasis. However, there has been limited progress in identifying biomarkers of chemotherapy drug resistance in patients with HSCC.

In the post-genetic era, proteins are an important participant involved in extensive biological processes, molecular function, and cellular component formation. High-throughput proteomics, *via* expression differences, can be applied to precisely analyze, and distinguish diagnostic and prognostic molecular markers. Proteomic screening is usually performed with the use of liquid chromatography-tandem mass spectrometry (LC-MS), which is based on data-independent acquisition (DIA). This method effectively identifies specifically targeted proteins and molecular markers. This is the first study to investigate the protein expression differences between samples sensitive and resistant to chemotherapy drugs from HSCC and paraneoplastic tissues in a protein cytology analysis.

## Materials and Methods

### Patient Samples and Characteristics

Twenty-five pairs of HSCC tissues were taken from the Shanghai General Hospital for surgery from 2016 to 2019. All patients were male, aged 48 to 80 years, with an average age of 70.0 years. Among them, 12 patients underwent two courses of induction chemotherapy before surgery. The chemotherapy regimen was: Erbitux (cetuximab) + loboplatin + 5-Fu. There were five patients with stage I–II, 19 patients with stage III–IV (including 1 patient who did not undergo surgery), 20 patients with piriform fossa, two patients with posterior pharyngeal wall, and three patients with posterior ring. All pathological sections were reviewed by two senior pathologists to confirm the diagnosis. Three cases were well-differentiated: 22 cases had medium-low differentiation, nine cases had no lymph node metastasis, and 15 cases had lymph node metastasis (see **Table 1**). The mucosa tissues of HSCC were normal tissue without hyperplasia and neoplastic lesions. All samples were stored at −80°C.

**Table 1:** Clinical data of 25 patients with HSCC.

Factor		Quantity (N%)
Gender	Male	25 (100%)
	Female	0
Age	<60	9 (36%)
	≥60	16 (64%)
Anatomical site	Posterior pharyngeal wall	3 (12%)
	Piriformis	20 (80%)
	Behind the ring	2 (8%)
Smoking	Yes	8 (32%)
	No	17 (68%)
Drinking	Yes	15 (60%)
	No	10 (40%)
Clinical stage	Phase 1 & 2	5 (25%)
	Phase 3 & 4	19 (76%)
T staging	T1	3 (12%)
	T2	12 (48%)
	T3	8 (32%)
	T4	1 (4%)
N staging	N0	9 (36%)
	N+	15 (60%)
Differentiation	Poorly differentiated	9 (36%)
	Medium differentiation	13 (52%)
	Highly differentiated	3 (12%)
Chemotherapy	No chemotherapy	13 (52%)
	Chemosensitivity	5 (20%)
	Chemotherapy resistance	7 (28%)

### Method

Sample preparation and fractionation for data dependent acquisition (DDA) library generation

Samples were first homogenized with a MP FastPrep-24 homogenizer (24 × 2, 6.0 M/S, 60 s, twice), and then SDT buffer (4% SDS, 100 mM DTT, 150 mM Tris-HCl pH 8.0) was added. The lysates were further sonicated (this step was omitted for protein solutions), and boiled for 15 min. After centrifuged at 14000 × g for 40 min, the supernatant was quantified with the BCA Protein Assay Kit (Bio-Rad, USA). The sample was stored at −80°C. An equal aliquot from each sample in this experiment was pooled into one sample for DDA library generation and quality control.

Protein digestion was performed according to the FASP procedure. Briefly, 200 μg of proteins were incorporated in 30 μL SDT buffer. The detergent, DTT, and other low-molecular-weight components were removed using UA buffer (8 M urea, 150 mM Tris-HCl pH 8.0) by repeated ultrafiltration (Microcon units, 30 kDa). Iodoacetamide (0.05 M, 100 μL) in UA buffer was added to block reduced cysteine residues; the samples were then incubated for 30 min in darkness. Filters were washed with 100 μL UA buffer three times and then 100 μL 25 mM NH4HCO3 twice. Finally, the protein suspensions were digested with 2 μg trypsin (Promega) in 40L 100 mM NH4HCO3 buffer overnight at 37°C. The resulting peptides were collected as a filtrate. Peptide content was estimated by UV light spectral density at 280 nm.

Digested pool peptides were then fractionated to ten fractions using High pH Reversed-Phase Peptide Fractionation Kit (Thermo Scientific™ Pierce™). Each fraction was concentrated by vacuum centrifugation and reconstituted in 15 µL of 0.1% (v/v) formic acid. Collected peptides were desalted on C18 Cartridges (Empore™ SPE Cartridges C18 standard density, bed I.D. 7 mm, volume 3 mL, Sigma) and reconstituted in 40 µL of 0.1% (v/v) formic acid.

The iRT-Kits (Biognosys) was added to correct the relative retention time differences between runs with volume proportion 1:3 for iRT standard peptides versus sample peptides.

#### DDA Mass Spectrometry Assay

All fractions for DDA library generation were injected on a Thermo Scientific Q Exactive HF mass spectrometer connected to an Easy nLC 1200 chromatography system (Thermo Scientific). Peptides (2 μg) were first loaded onto an EASY-SprayTM C18 Trap column (Thermo Scientific, P/N 164946, 3 μm, 75 μm * 2 cm), and then separated on an EASYSprayTM C18 LC Analytical Column (Thermo Scientific, ES803, 2 μm, 75 μm * 50 cm) with a linear gradient of buffer B (80% acetonitrile and 0.1% formic acid) at a flow rate of 250 nL/min over 120 min. MS detection method was positive ion, the scan range was 300–1650 m/z, and resolution for MS1 scan was 60000 at 200 m/z, target of automatic gain control (AGC) was 3e6, maximum IT was 25 ms, and dynamic exclusion was 30.0 s. Each full MS–SIM scan followed 20 ddMS2 scans. Resolution for MS2 scan was 15000, AGC target was 5e4, maximum IT was 25 ms, and normalized collision energy was 27 eV.

#### Mass Spectrometry Assay for DIA

Sample peptides were analyzed by LC-MS/MS operating in the DIA mode (Shanghai Applied Protein Technology Co., Ltd). Each DIA cycle contained one full MS–SIM scan, and 30 DIA scans covered a mass range of 350–1650 m/z with the following settings: SIM full scan resolution was 60,000 at 200 m/z, AGC 3e6, maximum IT 50 ms, profile mode, DIA scans were set at a resolution of 30,000, AGC target 3e6, Max IT auto, and normalized collision energy was 30 eV. Runtime was 120 min with a linear gradient of buffer B (80% acetonitrile and 0.1% formic acid) at a flow rate of 250 nL/min. QC samples (pooled sample from equal aliquot of each sample in the experiment) were injected with DIA mode at the beginning of the MS study and after every five injections throughout the experiment, which was used to monitor the MS performance.

#### Mass Spectrometry Data Analysis

For DDA library data, the FASTA sequence database was searched with MaxQuant software (version_1.5.3.17). The database was downloaded at website: http://www.uniprot.org. iRT peptides sequence was added (>Biognosys|iRTKit|Sequence_fusion LGGNEQVTRYILAGVENSKGTFIIDPGGVIRGTFIIDPAAVIRGAGSSEPVTGLDAK TPVISGGPYEYRVEATFGVDESNAKTPVITGAPYEYRDGLDAASYYAPVRADVT PADFSEWSKLFLQFGAQGSPFLK). The parameters were set as follows: the enzyme was trypsin, maximum of missed cleavages was 2, fixed modification was carbamidomethyl (C), and dynamic modifications were oxidation (M) and acetyl (Protein N-term). All reported data were based on 99% confidence for protein identification as determined by false discovery rate (FDR = N (decoy) * 2/(N (decoy) + N (target))) ≤ 1%. Spectral library was constructed by importing the original raw files and DDA searching results into Spectronaut Pulsar XTM_12.0.20491.4 (Biognosys).

DIA data was analyzed with Spectronaut Pulsar XTM searching the above constructed spectral library. Main software parameters were set: retention time prediction type was dynamic iRT, interference on MS2 level correction was enabled, and cross run normalization was enabled. All results were filtered based on Q value cutoff 0.01 (equivalent to FDR < 1%).

Bioinformatic analyses: The proteins were defined as differentially expressed if the fold-change between the disease groups and healthy controls was ≥ 2 or ≤ 0.5 and the P value < 0.05. The differentially expressed proteins were analyzed by hierarchical clustering to classify all samples (http://en.wikipedia.org/wiki/Cluster_analysis). Next, the differentially expressed proteins were subjected to gene ontology (GO) analysis by Blast2GO (https://www.blast2go.com/) and matched against the Kyoto Encyclopedia of Genes and Genomes (KEGG) database by the KEGG Automatic Annotation Server (KAAS, https://www.genome.jp/tools/kaas/). P < 0.05 in Fisher’s exact test was considered significant. Protein–protein interaction (PPI) networks were created for these proteins using the STRING database (http://string-db.org/).

ELISA validation for protein: To corroborate the expression of RIPK1 and FADD in HSCC, we validated their expression in neoplastic and paraneoplastic samples from 37 patients with HSCC using enzyme-linked immunosorbent assay (ELISA). ELISA kit was bought by Cell signaling technology company and according to its protocol. In accordance with clinical data, we analyzed the expression of RIPK1 and FADD proteins in 23 patients sensitive to chemotherapy vs 14 patients resistant to chemotherapy.

#### Equipment and Important Consumables

Q Exactive HF (Thermo Scientific), Easy nLC-1200 system (Thermo Scientific), Trap Column: Home-made column (100tific) steins in 2. Analytical Column: EasySpray Analytical Column (75 μm * 50 cm, 2.0 μm-C18), Spectronaut Pulsar X (12.0.20491.4), iRT Kit (Biogonosys).

#### Statistical Methods

We used SPSS software version 22.0 for Windows (IBM, Chicago, IL, USA) for statistical analyses. Data were assessed with the Shapiro–Wilk normality test. Continuous variables are expressed as mean ± standard deviations. Categorical variables are expressed as absolute numbers and percentages (%). The significance of the protein abundance changes was calculated using nonparametric Student’s t-test with Bonferroni multiple testing correction applied. A two-tailed test with P < 0.05 was considered significant. Graphs were prepared using GraphPad Prism version 7.0 (GraphPad Software, San Diego, CA, USA).

## Results

### Clinical Data of 25 Patients With HSCC

Twenty-five male patients with HSCC were recruited; sixteen were over 60 years old and nine were under 60 years old. One of them did not undergo surgical treatment after biopsy, so clinical staging, T staging, and N staging were not performed (see **Table 1**).

### Proteomics Analysis of HSCC

We used DIA to perform proteomic analysis on tumor samples and adjacent tissues of HSCC

**Figure 1:**
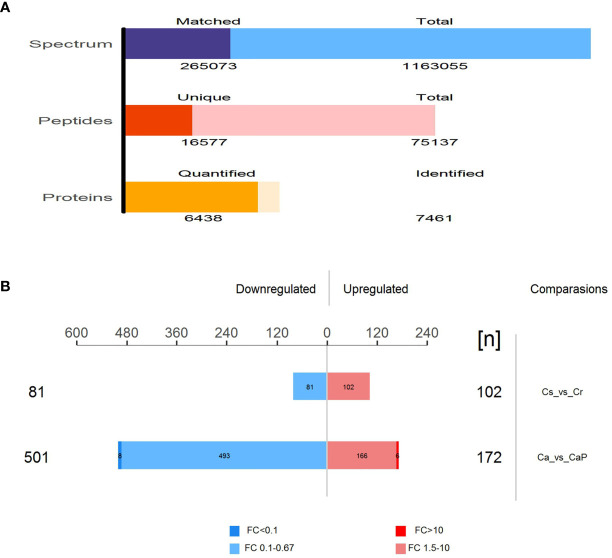
Total number of peptides and proteins identified by DIA database. **(A)** Total number of differentially expressed proteins in Ca_vs_CaP and Cs_vs_Cr groups. Quantitative values of proteins in the samples for subsequent statistical and bioinformatics analysis, including 6,438 proteins and 16,577 peptides. **(B)** Among the Ca_vs_CaP group, we screened 673 differentially expressed proteins (172 upregulated and 501 downregulated). A total of 183 differentially expressed proteins were screened in the Cs_vs_Cr group (102 upregulated and 81 downregulated).

The mixed samples of all samples used DDA mass spectrometry data acquisition method to establish the DIA library database of this project (**Table 2**). A total of fifty samples identified 251,169 proteins; 121,947 proteins were identified adjacent to cancer, and 129,222 proteins in cancer tissues were identified. To ensure the validity and accuracy we undertook follow-up biological and statistical analysis. The correlation coefficient of QC samples indicated the stability of the entire experimental operation and the reliability of the test results. In this study, we selected proteins at constant levels found in >50% samples for subsequent statistical and bioinformatic analyses, including 6,438 proteins and 16,577 peptides. Among the Cancer vs ParaCancer (Ca_vs_CaP) group, we screened 673 differentially expressed proteins (172 upregulated and 501 downregulated). A total of 183 differentially expressed proteins were screened in the Chemo-sensitive vs Chemo-resistant (Cs_vs_Cr group) (102 upregulated and 81 downregulated) (see **Figure 1**).

**Table 2:** DIA protein identification results statistics.

Sample (Ca Group)	Protein groups	Sample (CaP Group)	Protein groups
Ca81	4712	CaP81	4970
Ca90	5468	CaP90	5336
Ca126	5531	CaP126	5124
Ca137	4427	CaP137	4720
Ca147	5539	CaP147	4613
Ca164	5460	CaP164	5271
Ca165	5573	CaP165	4909
Ca172	5164	CaP172	4964
Ca179	5374	CaP179	5215
Ca184	5076	CaP184	5181
Ca194	5132	CaP194	4592
Ca196	5410	CaP196	5596
Ca204	4091	CaP204	4861
Ca236	5097	CaP236	4801
Ca257	5303	CaP257	3788
Ca273	5192	CaP273	4186
Ca297	5345	CaP297	5020
Ca304	5273	CaP304	5095
Ca306	5369	CaP306	5378
Ca314	5065	CaP314	5051
Ca339	5262	CaP339	5214
Ca353	5395	CaP353	5352
Ca360	4093	CaP360	3777
Ca363	5385	CaP363	5308
Ca389	5486	CaP389	3625

### Differential Protein Analysis

**Figure 2:**
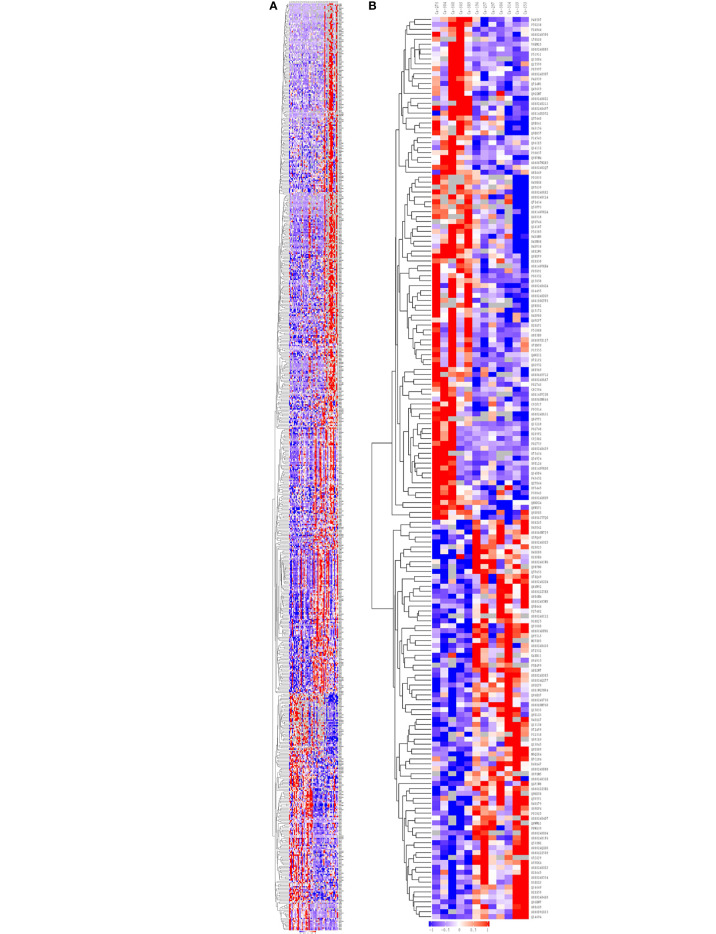
Cluster analysis of differentially expressed proteins in the group of Ca_vs_CaP and Cs_vs_Cr. The hierarchical clustering results are represented as a tree heat map, with the ordinate representing significantly differentially expressed proteins and the abscissa representing sample information. Significant differences in protein expression in the different numerical expression quantities (log2 expression) of the samples with different colors are shown in the heat map, where red represents significantly upregulated proteins, blue represents significantly downregulated proteins, and gray represents no quantitative information for proteins. **(A)** Ca_vs_CaP. **(B)** Cs_vs_Cr. Ca273, Ca304, Ca360, Ca363, and Ca389 are in the Cs group. Ca196, Ca257, Ca297, Ca306, Ca314, Ca229, and Ca353 are in the Cr group. FADD equals to the protein name of Q13158. RIPK1 equals to the protein name of A0A024QZU0. Both of them were marked by yellow block.

Hierarchical clustering algorithm (Hierarchical Cluster) was used to perform cluster analysis on the differentially expressed proteins of the comparison group, and the data was displayed in the form of heat map (Heatmap), as shown in **Figure 2**.

Hierarchical clustering analysis was conducted on 673 and 183 dysregulated proteins, and the heatmap obtained from the analysis provided protein profiles for Ca_vs_CaP and Cs_vs_Cr (**Figure 2**).

Joint analysis of 39 differentially expressed proteins in the Ca_vs_CaP and Cs_vs_Cr groups, combined with known protein functions, found that 21 protein biological behaviors were related to tumors. Further screening for proteins related to the tumor chemotherapy sensitivity, inspiringly, gene FADD and its role-related genes RIPK1, found that FADD in the Ca group was highly expressed, and RIPK1 was poorly expressed in the Ca group. The expression of FADD and RIPK1 in the chemotherapy-sensitive group were significantly lower than those in the chemotherapy-resistant group. We analyzed the expression of FADD and RIPK1 between the chemotherapy-sensitive and non-chemotherapy groups, and between the chemotherapy-resistant and non-chemotherapy groups and found that there was no significant difference in FADD expression. The expression of RIPK1 in the chemotherapy-sensitive group was lower than that of the non-chemotherapy group (**Table 3**).

**Table 3:** Expression of FADD and RIPK1 in different groups of hypo pharyngeal carcinoma.

	Ca/CaP	T-test P value	Cs/Cr	T-test P value	Cs/Noc	T-test P value	Cr/Noc	T-test P value
FADD	1.6964	0.0378*	0.257	0.016*	0.3222	0.0637	1.2537	0.4766
RIPK1	0.5048	0.001**	0.3887	0.0498*	0.4255	0.0458*	1.0947	0.7399

Ca/CaP, ratio of cancer/paraneoplastic group; Cs/Cr, ratio of chemotherapy-sensitive/chemo resistant group; Cs/Noc, ratio of chemotherapy-sensitive/non-chemotherapy group; ratio of chemotherapy-resistant/non-chemotherapy group; *P < 0.05; **P < 0.01.

### GO Annotation and Enrichment Analysis

**Figure 3:**
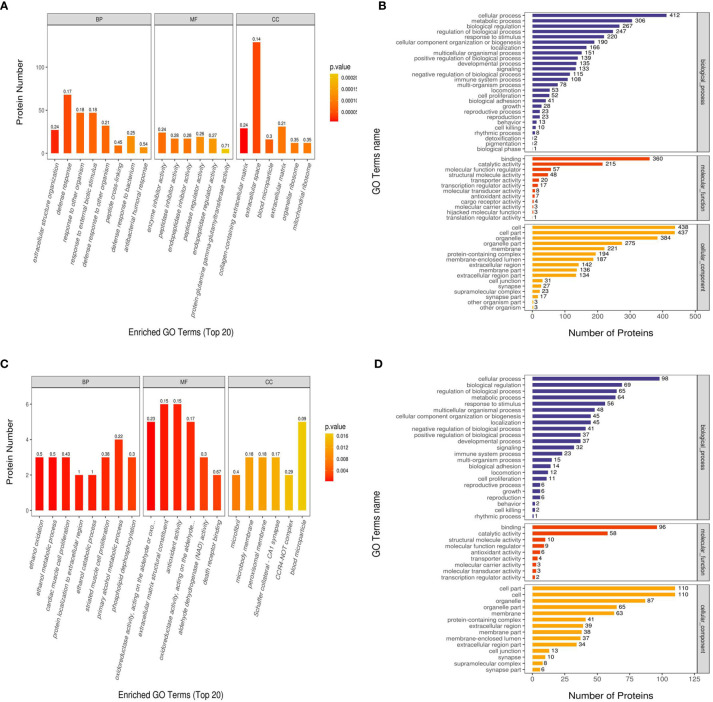
Gene Ontology (GO) functional enrichment analysis of Ca_vs_CaP and Cs_vs_Cr differentially expressed proteins. The abscissas in **(A, C)** represent the enriched GO functional classification, which is divided into three major categories: Biological Process (BP), Molecular Function (MF), and Cellular Component (CC). The ordinate indicates the number of differential proteins under each functional classification; the color of the bar graph indicates the significance of the enriched GO functional classification, which is based on Fisher’s accuracy; Fisher’s Exact Test calculated the P value. The color gradient represents the size of the P value, from orange to red; the closer to red, the smaller the P value, and the higher the significance level of the enrichment of the corresponding GO function category. The label above the bar graph shows the enrichment factor (Rich factor ≤ 1). The enrichment factor represents the ratio of the number of differentially expressed proteins annotated to a GO functional category to the number of all identified proteins annotated to the GO functional category. **(B, D)** show the number of differentially expressed proteins enriched in each entry.

We performed GO functional annotation on all the proteins screened in this project. In the Ca_vs_CaP group, the difference in protein expression was greatest in Biological Process (BP), Molecular Function (MF), and Cellular Component (CC). Concentrated results were in defense response, enzyme inhibitor activity, and extracellular space. In the Cs_vs_Cr group, the most concentrated protein expression differences with BP, MF, and CC were in primary alcohol metabolic process, antioxidant activity, and blood micro particle (**Figure 3**). RIPK1 and FADD are mainly enriched in the BP necroptotic signaling pathway and MF death receptor binding.

### KEGG Annotation and Enrichment Analysis

**Figure 4:**
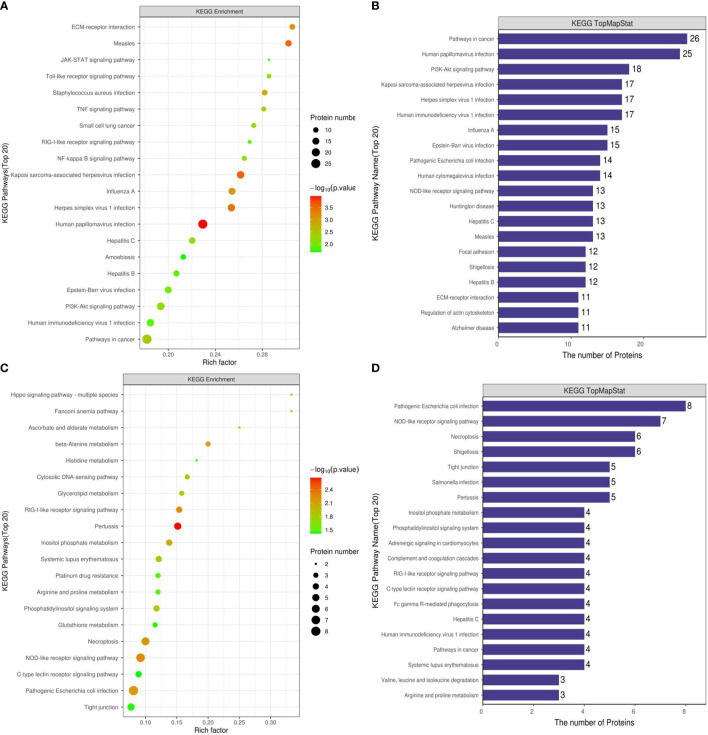
KEGG functional enrichment analysis of Ca_vs_CaP and Cs_vs_Cr differentially expressed protein signaling pathways. **(A, B)** are Ca_vs_CaP differentially expressed proteins mainly concentrated in HPV infection, pathway in cancer, and PI3K-Akt signaling pathway. **(C, D)** are Cs_vs_Cr differentially expressed proteins mainly concentrated in pathogenic Escherichia coli infection, NOD-like receptor signaling pathway, and necroptosis RIPK1 and FADD proteins were mainly present in necroptosis and PRRs (containing NOD-like receptor signaling pathway, RIG-I-like receptor signaling system, and Toll-like receptor signaling pathway).

We used KEGG to analyze the signaling pathways of 673 differentially expressed proteins in the Ca_vs_CaP group that were mainly concentrated in HPV infection, pathway in cancer, and PI3K-Akt signaling pathway. The signaling pathways where183 differentially expressed proteins of the Cs_vs_Cr group were mainly concentrated in were pathogenic Escherichia coli infection, NOD-like receptor signaling pathway, and necroptosis (**Figure 4**). RIPK1 and FADD proteins were mainly present in necroptosis and pattern-recognition receptors (PRRs) (containing NOD-like receptor signaling pathway, RIG-I-like receptor signaling system, and Toll-like receptor signaling pathway).

### Protein Interaction Analysis

**Figure 5:**
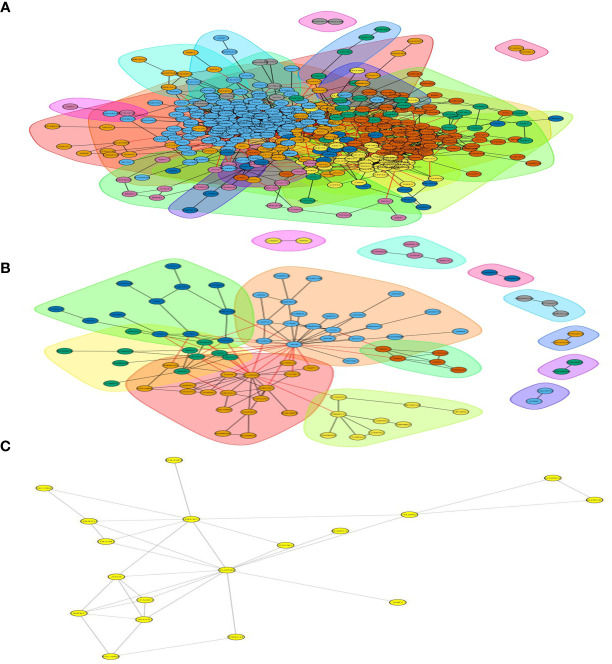
Differentially expressed protein interaction networks in group of Ca_vs_CaP and Cs_vs_Cr. In the protein interaction network, nodes represent proteins and lines represent protein–protein interactions. Yellow nodes are differentially expressed proteins. The number of proteins directly interacting with protein A is called the linkage degree of the protein. **(A)** Differentially expressed protein interaction networks in group of Ca_vs_CaP. **(B)** Differentially expressed protein interaction networks in group of Cs_vs_Cr. **(C)** FADD and RIPK1 involved interacting protein.

From the 673 differentially expressed proteins in the Ca_vs_CaP group, 432 proteins were involved in protein interactions, and 93 of the 183 differentially expressed proteins in the Cs_vs_Cr group involved protein interactions (**Figure 5**).

Both RIPK1 and FADD involved 12 interacting proteins in the Ca_vs_CaP group; RIPK1 involved five interacting proteins in the Cs_vs_Cr group, and FADD involved four interacting proteins in the Cs_vs_Cr group (**Figure 5**).

### ELISA Analysis

**Figure 6:**
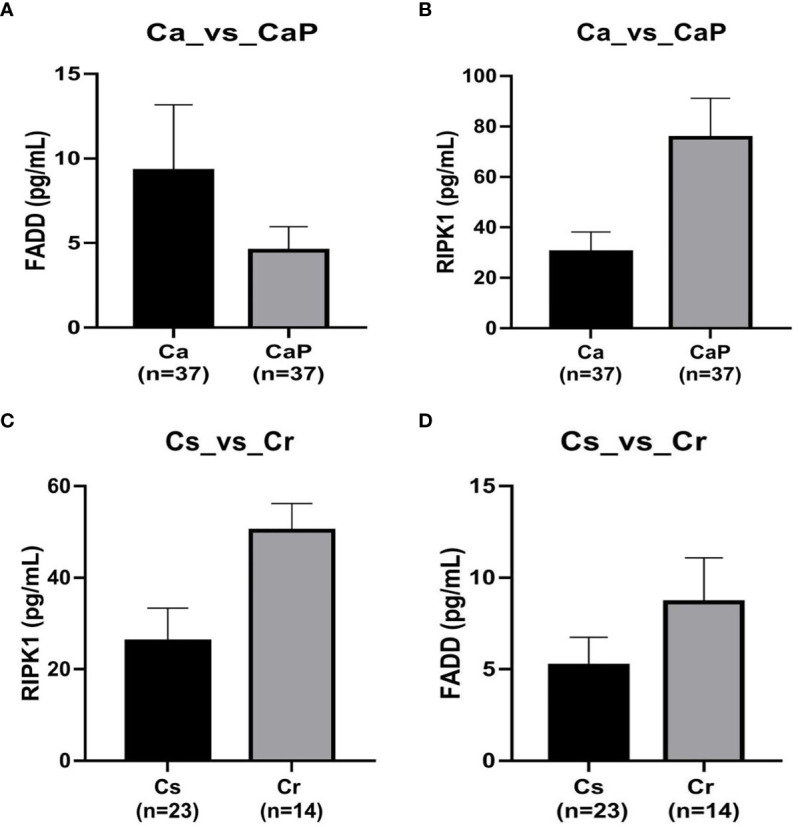
Verification by ELISA of expression of FADD and RIPK1 proteins in hypopharyngeal squamous cell carcinoma (HSCC) samples. **(A)** in tissue adjacent to cancer, FADD is more highly expressed in 37 cases of HSCC, P < 0.05. **(B)** whereas RIPK1 is poorly expressed, P < 0.05. **(C)** FADD expression was lower in 23 cases of chemotherapy-sensitive patients compared with expression in 14 cases of chemotherapy-resistant patients, P < 0.05. **(D)** RIPK1 expression was lower in 23 chemotherapy-sensitive patients compared with 14 cases of chemotherapy-resistant patients, P < 0.05.

We used ELISA to verify the expression of RIPK1 and FADD proteins in the HSCC tumors and adjacent samples of 37 patients with HSCC, and analyzed 23 patients who were chemotherapy-sensitive and 14 patients with chemotherapy resistance, according to clinical data. The results show that FADD is highly expressed in HSCC, whereas RIPK1 is poorly expressed, and both RIPK1 and FADD are poorly expressed in chemotherapy-sensitive patients (**Figure 6**).

## Discussion

HSCC is not readily detected and is diagnosed by a shaded anatomical site and the absence of typical symptoms in the early stage. It is also characterized by the tendency of submucosal dissemination and localized lymph node metastasis. Therefore, approximately 80% of patients are diagnosed at the middle-to-advanced stages, with a poor prognosis (10). Comprehensive therapy is now the primary treatment for HSCC, particularly at an advanced stage, which includes chemotherapy as an essential treatment choice (11). We previously conducted a single-center, randomized, double-blinded clinical study in patients with HSCC. The induction chemotherapy group was treated in combination with surgery and radiotherapy: Erbitux (cetuximab), lobaplatin, and 5-Fu while the control group received surgery and radiotherapy. The induction chemotherapy group showed significant increases in overall and partial response rates as well as elevations in larynx preservation, patient life quality, and 5-year survival rate compared with these factors in control group. However, 40.62% of patients were still insensitive to targeted or chemotherapy drugs or appeared sensitive initially but subsequently became resistant to drugs, and did not achieve remission with cases of disease progression. Thus, platinum-based and anti-EGFR targeted drugs still have significant shortcomings, such as multiple drug resistance, which severely limits their efficacy.

The growth in proteomics techniques (especially DIA-based LC-MS/MS) demonstrates their enormous advantage in screening for tumor molecular markers (12). Coscia et al. (13) conducted a proteomic analysis in patients with advanced ovarian cancer, including 14 patients with sensitivity to chemotherapy and 11 patients with resistance to chemotherapy. They found that cancer-testis antigen 45 (CT45) is an independent prognostic factor associated with double prolongation of disease-free survival in high-grade serous ovarian cancer (HGSOC); CT45 thus acts as a novel prognostic indicator of HGSOC, a regulatory site of sensitivity to chemotherapy, and a target of immunotherapy. Proteomic screening for molecular markers of HSCC, particularly those of resistance to chemotherapy, has not been reported. In this study, there were a total of 673 differentially expressed proteins, including upregulated 172 and downregulated 501 proteins from HSCC samples, as compared with those from paraneoplastic samples. In patients resistant to chemotherapy, there were a total of 183 differentially expressed proteins, including upregulated 102 and downregulated 81 ones compared to those from patients sensitive to chemotherapy; both groups had 39 differentially co-expressed proteins. After analyzing the biological behavior of all of the 39 proteins, inspiringly, FAS-associated death domain-containing protein (FADD) was highly expressed in the tumor, indicating its role in the biological behavior of HSCC. At the same time, it was also poorly expressed in chemotherapy-sensitive patients and highly expressed in chemotherapy-resistant patients, further indicating that FADD may play a role in tumor chemotherapy sensitivity.

FADD was initially described as an adaptor molecule for death receptor-mediated apoptosis and was subsequently implicated with nonapoptotic cellular processes. In the last decade, FADD has appeared with new roles in innate immunity, inflammation, and cancer development (14). FADD is an essential molecule of the Fas/FasL apoptotic system and is also associated with the modulation of cell cycle. Although this study demonstrated significantly higher expression of FADD in HSCC tissue than in paraneoplastic tissue, the expression level was not statistically different in terms of the key patient symptoms such as primary site, T staging, N staging, the presence of lymph node metastasis, and degree of tumor differentiation, or whether the patient was a smoker or non-smoker. Therefore, it can be seen that FADD is most likely to play a role in the chemotherapy resistance of hypopharyngeal cancer, which is quite consistent with the conclusion drawn by our proteomics, which also fully arouses our interest in FADD.

Receptor Interacting Protein Kinase 1(RIPK1) (15) contains a kinase domain for Ser/Thr specificity at its N-terminus, which catalyzes phosphorylation of RIP at the Ser/Thr residue site; a death domain at its C-terminus, which interacts with members of the death receptor family, Fas and tumor necrosis factor receptor 1 (TNFR1) [i.e., a combination with TNF-associated death domain (TRADD) and FADD], and an intermediate domain between its N-terminus and C-terminus, including a RIP homotypic interaction motif that mediates homotypic and RIPK1-RIPK3 interactions (16). As a combination of RIPK1 and FADD, the FADDosome has emerged as an important factor in autophagy, tumor growth promotion, and resistance to chemotherapy (17, 18). Compared with paraneoplastic tissue, significantly lower expression of RIPK1 in HSCC tissue was the cause of resisting cell death in patients with HSCC. We further analyzed the expression of FADD and RIPK1 in chemotherapy sensitivity group vs. non-chemotherapy group (Cs_vs_Noc) and in chemotherapy resistance group vs. non-chemotherapy group (Cr_vs_Noc). No statistical difference in FADD expression was noted, and the expression of RIPK1 was lower in the sensitivity group than in the non-chemotherapy group. Compared with the chemotherapy resistance group, the lower expression of both RIPK1 and FADD in the chemotherapy sensitivity group indicated that high expression of RIPK1 and FADD resulted in resistance to chemotherapy.

GO enrichment analysis showed that RIPK1 and FADD contributed to chemotherapy resistance *via* cellular processes (necroptotic signaling pathway and regulation of necroptotic process) and molecular function of death receptor binding. In organisms, an individual protein does not work alone but coordinates biochemical responses with other proteins to establish their biologic function. Pathway analysis is the most effective method to understand biologic processes, cell traits, disease pathologies, and drug mechanisms of action in a combined fashion. Pathway analysis with KEGG was carried out with significantly differentially expressed proteins from the Cs_vs_Cr groups and demonstrated that RIPK1 and FADD are involved in necroptosis and the signaling pathway of PRRs. These findings are consistent with conclusions drawn by Roy (19). who found that nearly 30% of HNSCCs overexpressed FADD, and that FADD was a critical component of the TNFR signaling pathways, with or without BIRC2/3 genes encoding cellular inhibitor of apoptosis proteins 1/2 (cIAP1/2). A publication by Derakhshan (20) demonstrated that cell lines containing FADD amplifications displayed increased sensitivity to inhibitor of apoptosis proteins (IAP) antagonism while FADD overexpression sensitized a previously resistant, low-FADD expressing HNSCC cell line to birinapant and TNFα. This revealed that FADD expression varied with types of HNSCCs, and thus may show very different effects. Chemotherapy with additional SMAC mimetics and IAP antagonists was presumed to improve efficacy and reverse resistance among patients resistant to chemotherapy drugs. Differences in expression of RIPK1 and FADD were found in all three signaling pathways of PRRs, including those of Toll-like receptors, RIG-I-like receptors, and NOD-like receptors. The signaling pathways of PRRs have been implicated in many illnesses ranging from infection susceptibility to cancer and autoimmune disease (21). This finding shows the involvement of RIPK1 and FADD in immunomodulation associated with HSCC to induce chemotherapy resistance. The specific mechanism of action remains to be further examined.

To validate the low expression of RIPK1 and FADD in chemotherapy sensitivity for HSCC as revealed in the proteomic analysis, we evaluated expression of RIPK1 and FADD *via* ELISA in neoplastic and paraneoplastic samples from 37 patients with HSCC, including 23 patients sensitive to chemotherapy and 14 patients resistant to chemotherapy. The finding was consistent with the proteomic analysis, illustrated by the fact that RIPK1 and FADD showed high expression in neoplastic tissue and low expression in patients sensitive to chemotherapy. RIPK1 and FADD can therefore be considered as a predictor of resistance to chemotherapy for HSCC.

## Conclusions

LS-MC is an effective approach to predict tumor-associated markers. FADD can be considered as a molecular marker for predicting sensitivity to chemotherapy for HSCC because of the high expression in HSCC tissue and low expression in patients sensitive to chemotherapy. The RIPK1/FADD complex possibly exerts its significant effect by necroptosis and the signaling pathway of PRRs in patients with HSCC who are resistant to chemotherapy drugs, it need further research to prove.

## Data Availability Statement

The datasets presented in this study can be found in online repositories. The names of the repository/repositories and accession number(s) can be found in the article/supplementary material.

## Ethics Statement

The studies involving human participants were reviewed and approved by Shanghai General Hospital. The patients/participants provided their written informed consent to participate in this study.

## Author Contributions

Conceptualization, BS and XD. Methodology, BS. Software, BY. Formal analysis, BS. Investigation, BS. Writing—original draft preparation, BS. Writing—review and editing, ZZ. Supervision, ZZ. Project administration, ZZ. All authors contributed to the article and approved the submitted version.

## Conflict of Interest

The authors declare that the research was conducted in the absence of any commercial or financial relationships that could be construed as a potential conflict of interest.

## Abbreviations

HSCC, Hypopharyngeal squamous cell cancer; MDR, Multidrug Resistance; FADD, FAS-associated death domain-containing protein; RIPK1, Receptor Interacting Protein Kinase 1; LS-MC, liquid chromatography-tandem mass spectrometry.
